# Supplementary material for: The effect of myalgic encephalomyelitis/chronic fatigue syndrome (ME/CFS) severity on cellular bioenergetic function
Source: PLoS One. 2020 Apr 10;15(4):e0231136. doi: 10.1371/journal.pone.0231136 (PMC7147788; doi:10.1371/journal.pone.0231136)

**Supporting information – S1 appendix**

Multi-variate analysis was used to investigate the relationship between different parameters from the mitochondrial stress test conducted using PBMCs from healthy controls (n=12), moderately affected ME/CFS patients (n=13), and severely affected ME/CFS patients (n=25). Graphs for the correlations are shown below.


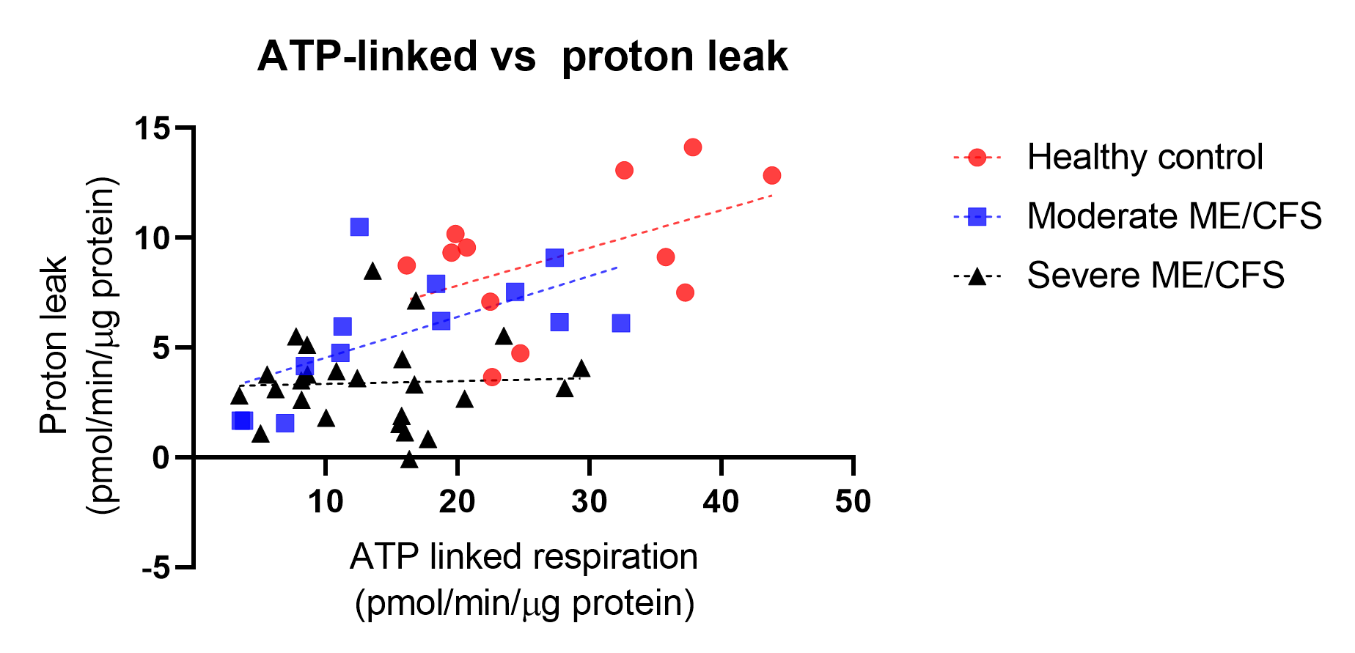

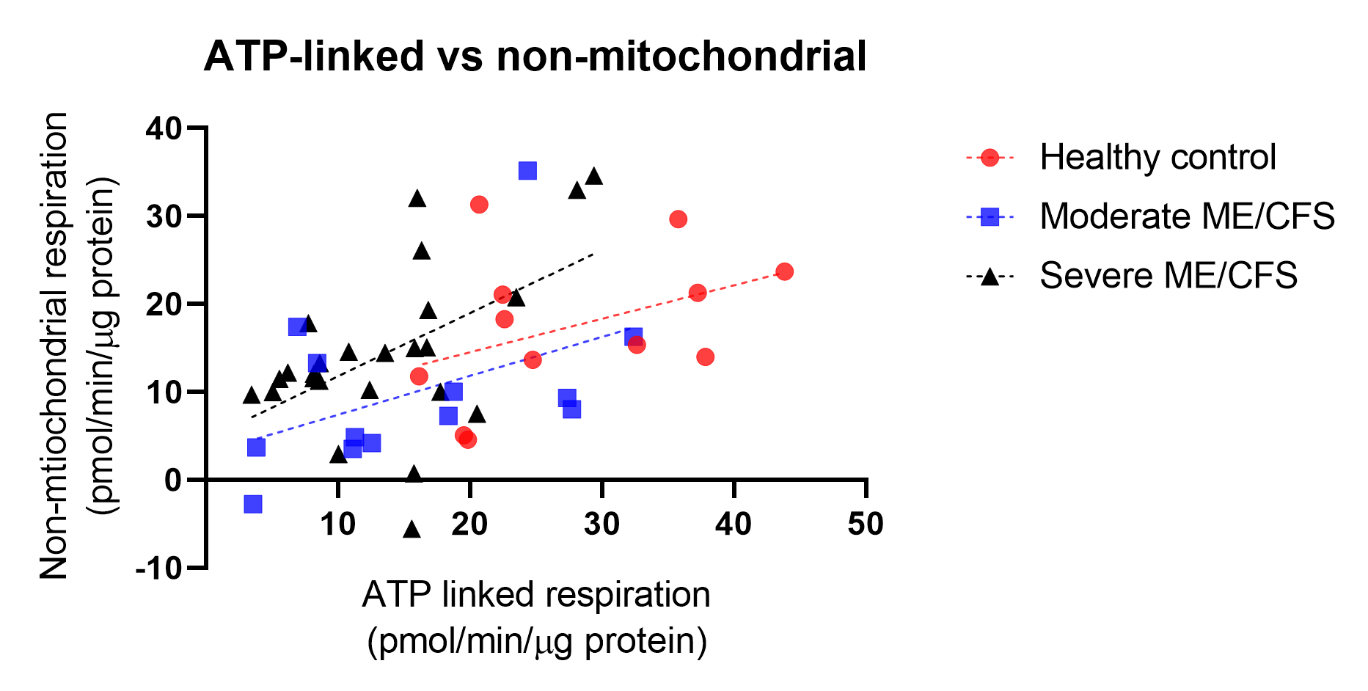

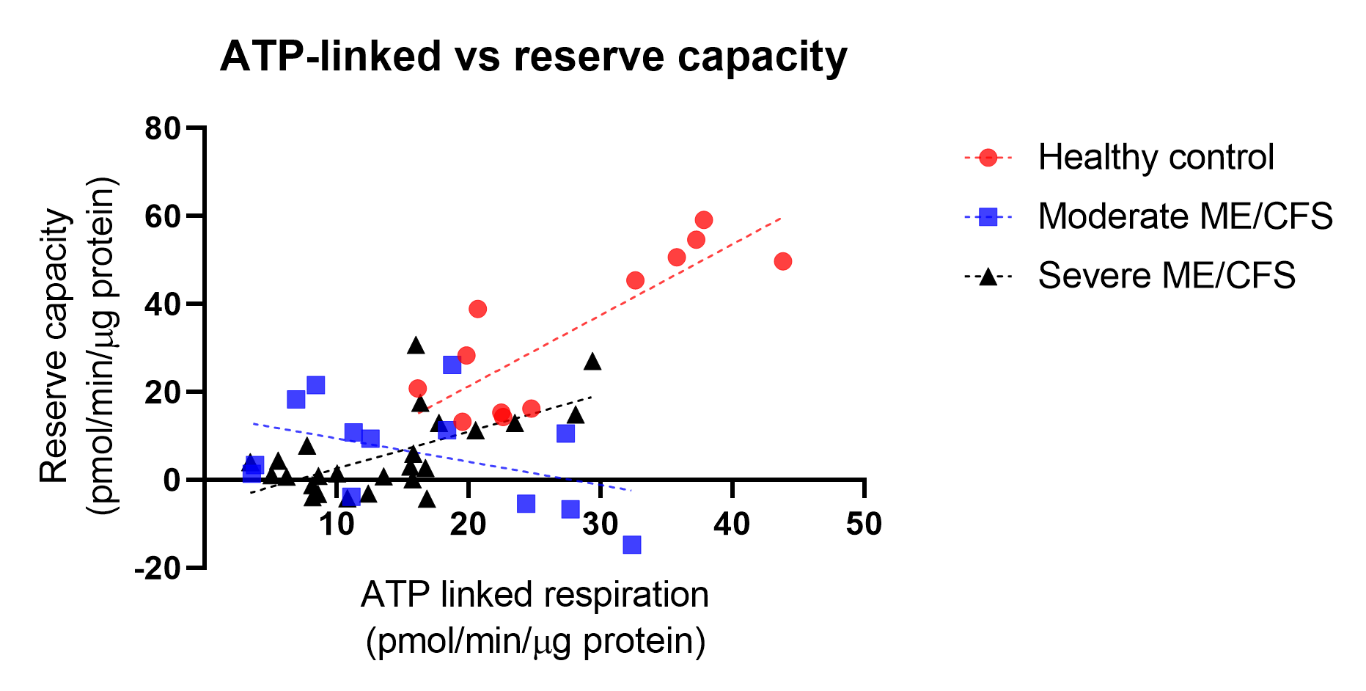

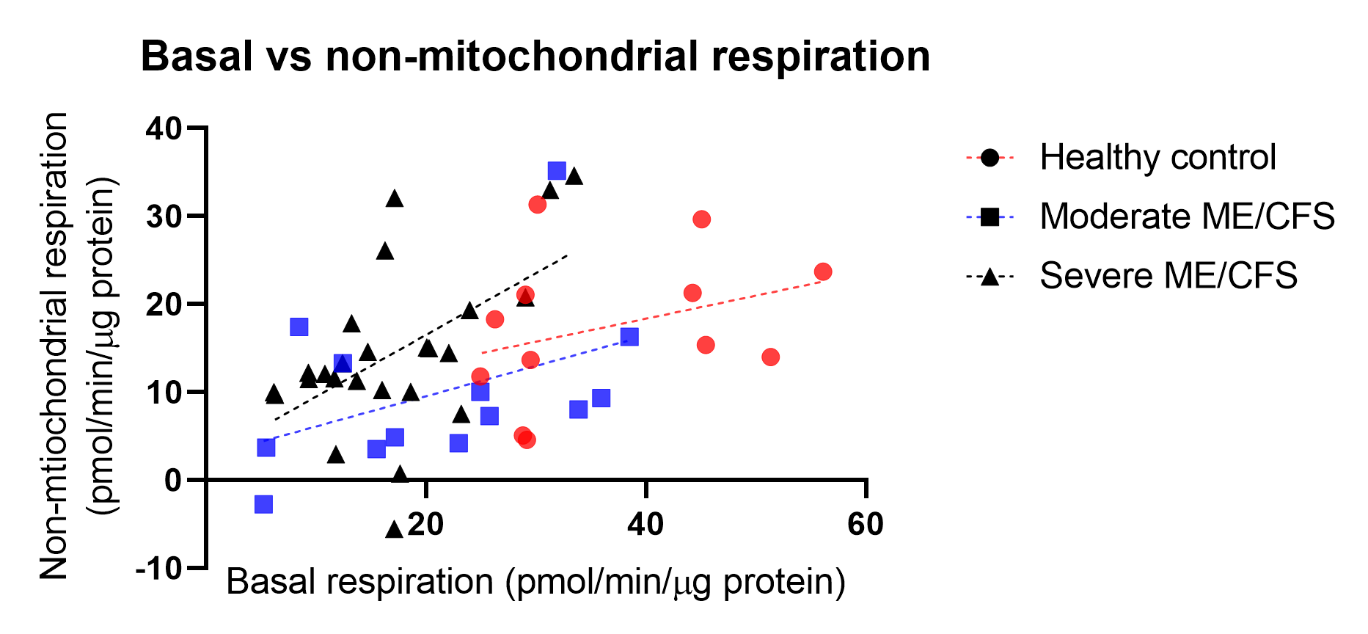

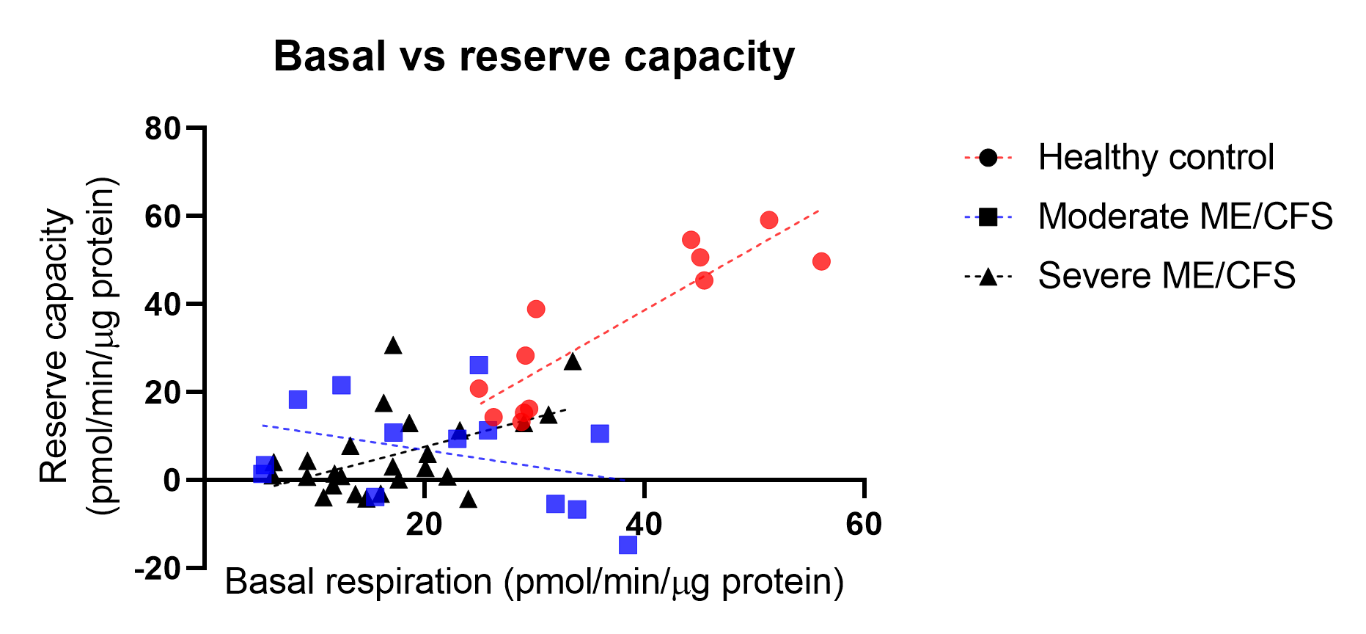

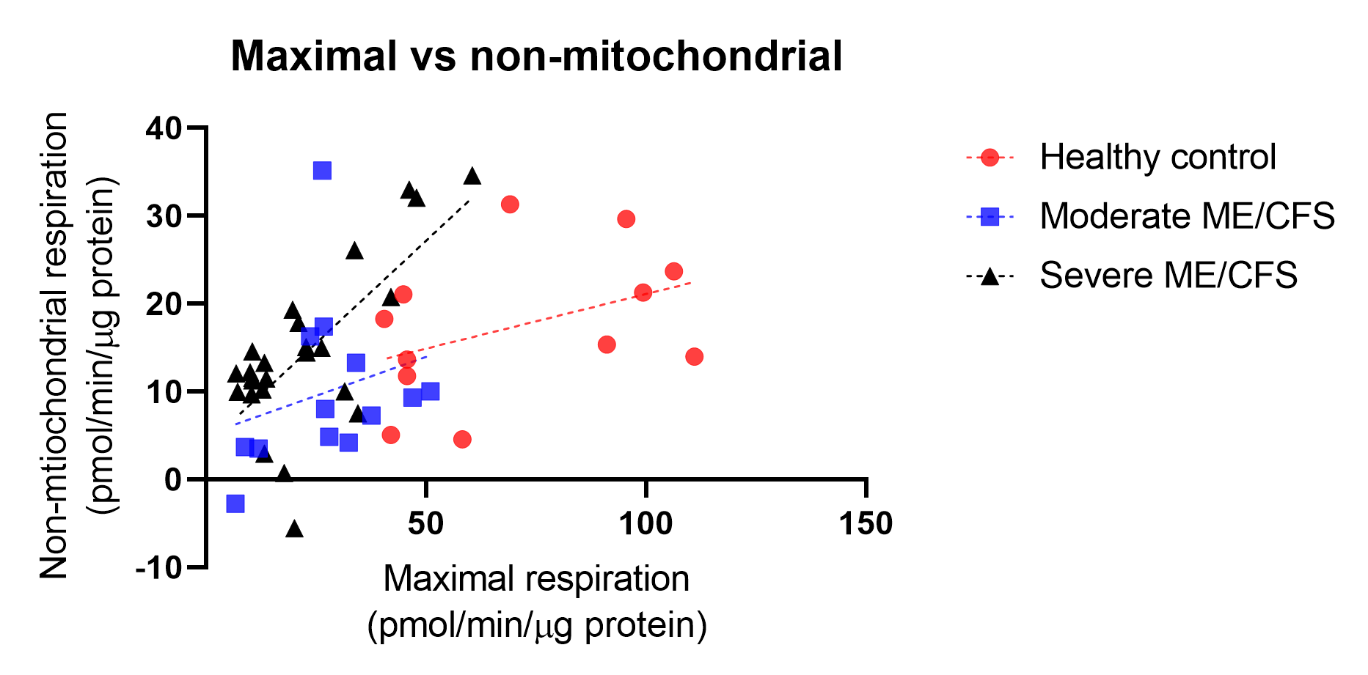

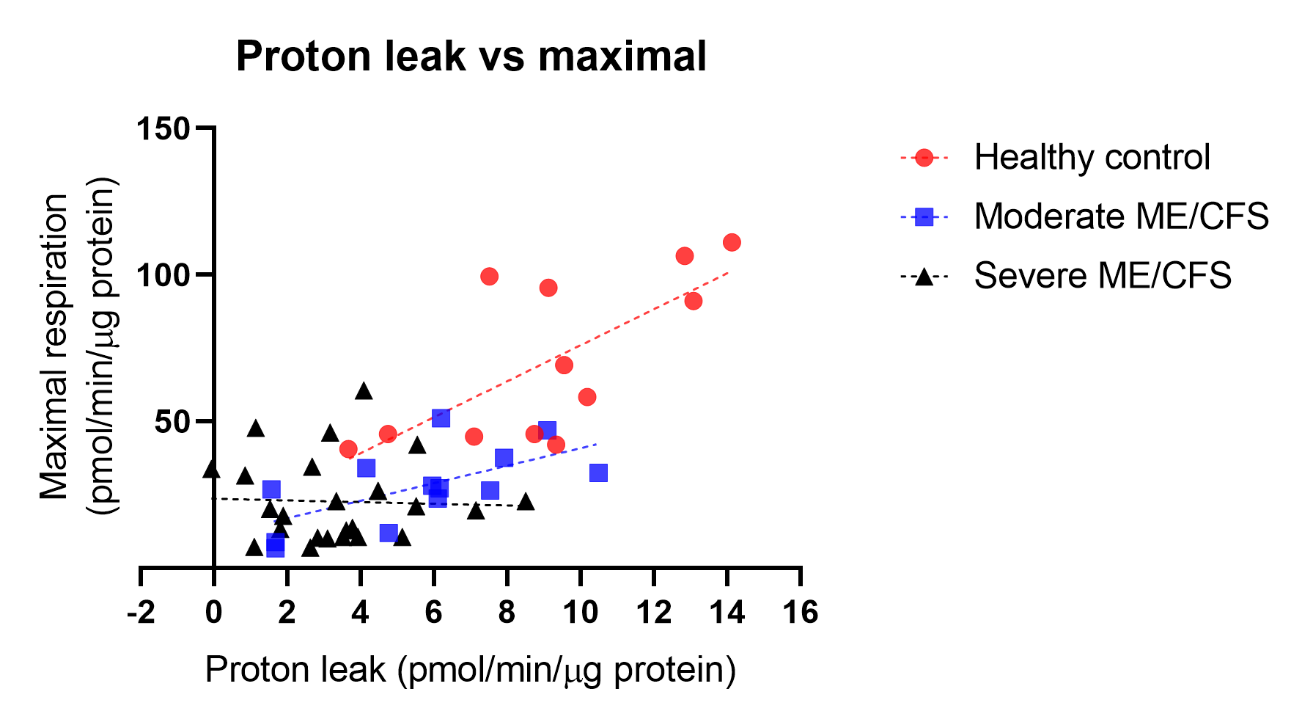

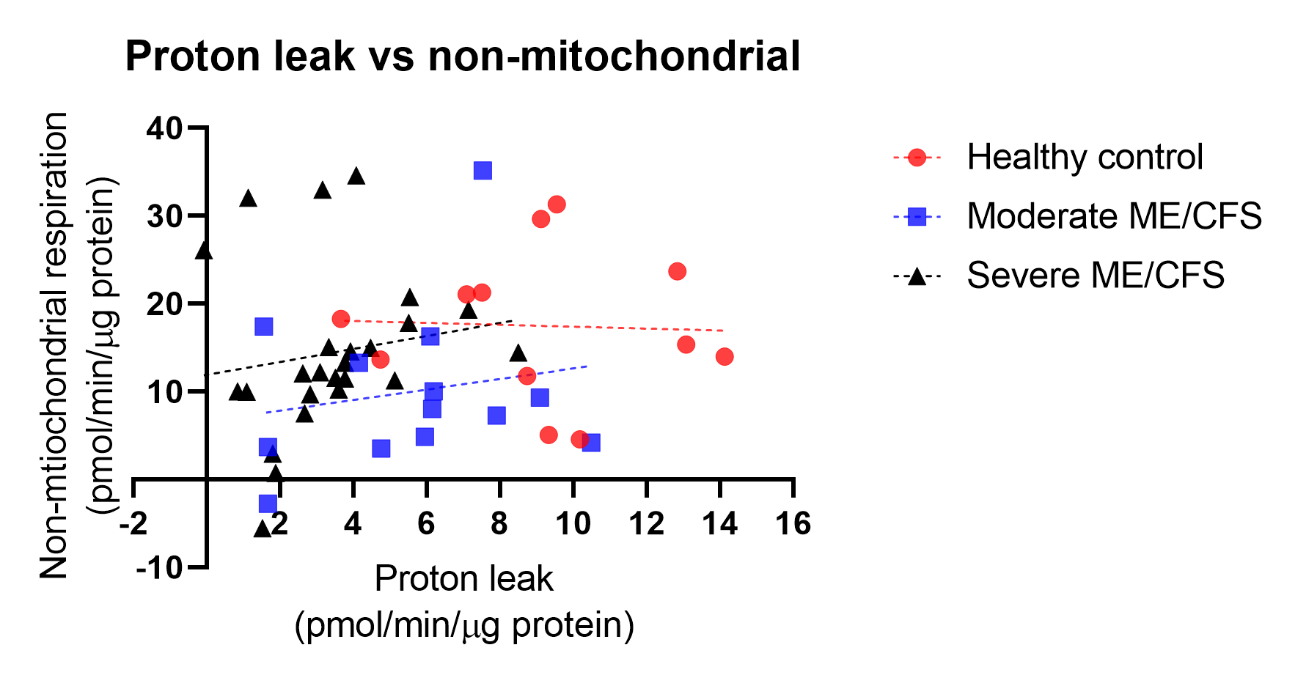

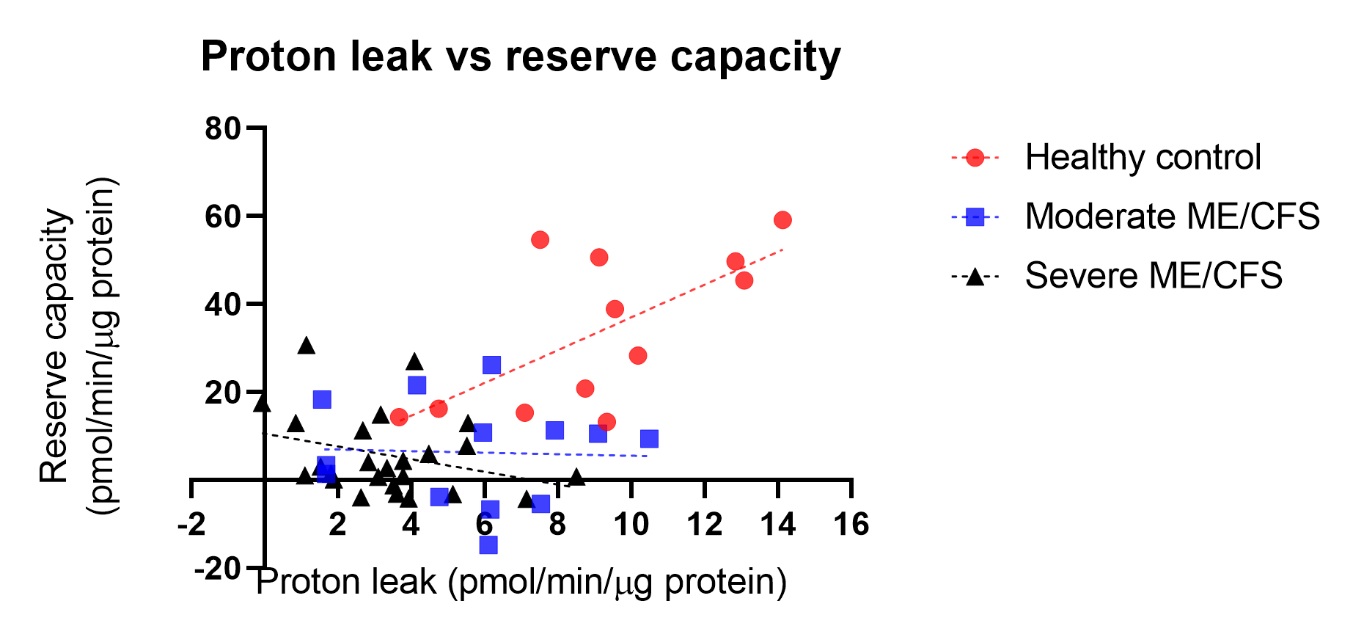

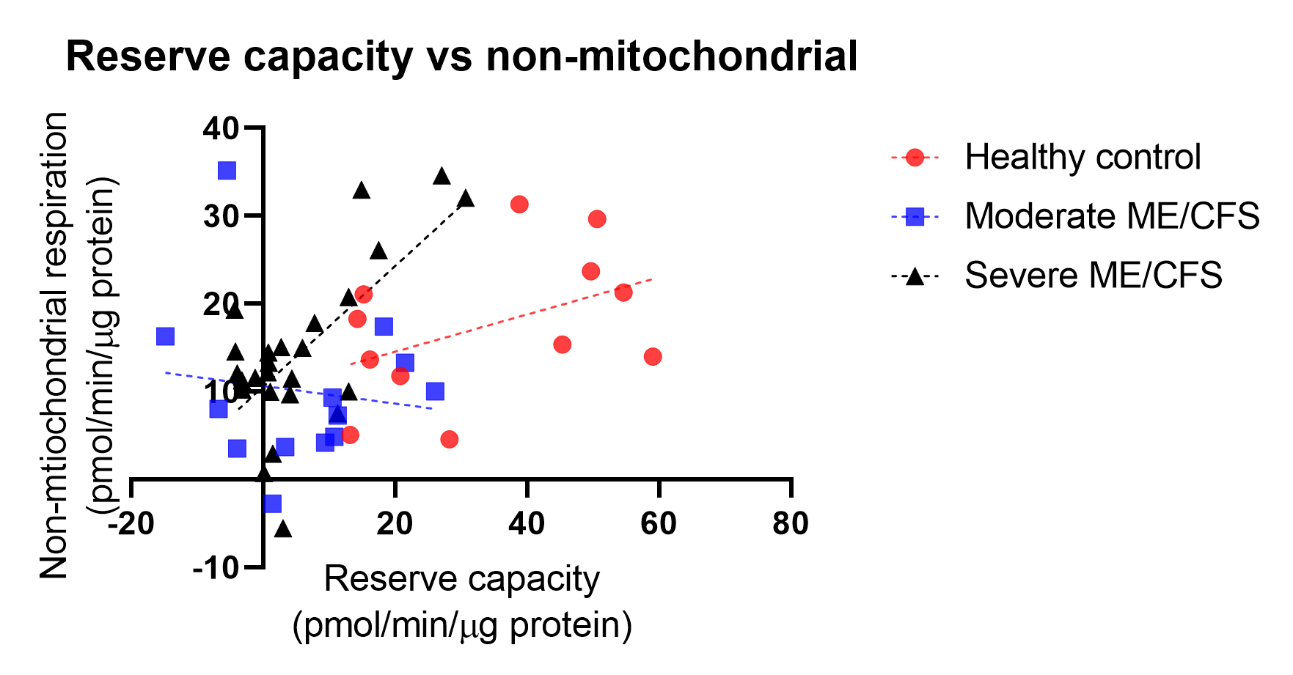

Supplement: S1 Appendix — (DOCX) [file pone.0231136.s001.docx]
