# Supplementary material for: The effect of myalgic encephalomyelitis/chronic fatigue syndrome (ME/CFS) severity on cellular bioenergetic function
Source: PLoS One. 2020 Apr 10;15(4):e0231136. doi: 10.1371/journal.pone.0231136 (PMC7147788; doi:10.1371/journal.pone.0231136)
Supplement: S2 Appendix — These graphs show the rates of glycolysis, glycolytic capacity, glycolytic reserve, and non-glycolytic acidification when respiratory acidification had been taken into account. (DOCX) [file pone.0231136.s002.docx]

**Supporting information – S2 appendix**

**Glycolytic parameters adjusted for respiratory acidification**

Data showing parameters of glycolytic respiration published by Tomas et al (2017) were re-analysed in order to take into account respiratory acidification of the cells. There were no significant differences between in the PBMCs from the ME/CFS cohort as a whole (n=19) and the healthy control cohort (n=16) for any of the glycolytic parameters calculated from a glycolysis stress test (p≥0.263) (Figure S1).


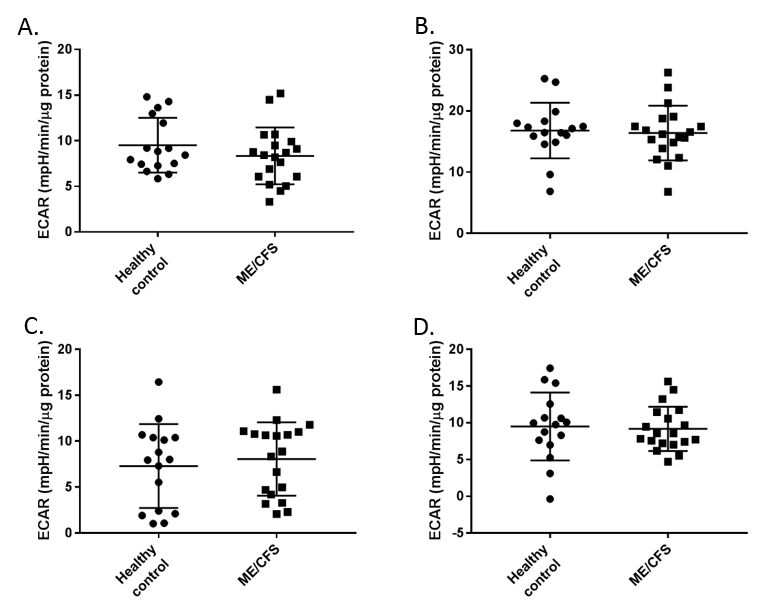


**Figure S1.** Glycolytic parameters for healthy control (n=16) and ME/CFS (n=19) PBMCs re-calculated after taking into account respiratory acidification. (A) Glycolysis. (B) Glycolytic capacity. (C) Glycolytic reserve. (D) Non-glycolytic acidification. Groups were compared using student’s t-tests with post-hoc Bonferroni correction.

**Glycolytic parameters of healthy control, moderate ME/CFS, and severe ME/CFS cohorts pre- and post-adjusting for respiratory acidification**

The ME/CFS cohort used by Tomas et al (2017) was separated out into moderately and severely affected group in order to see if disease severity affected parameters of glycolytic function in PBMCs. Healthy control, moderate ME/CFS, and severe ME/CFS groups were compared both before and after adjusting for respiratory acidification. The three parameters analysed (in addition to glycolysis which is included in the main manuscript) were glycolytic capacity (Figure S2), glycolytic reserve (Figure S3), and non-glycolytic acidification (Figure S4). There were no significant differences between any of the groups either before or after taking into account respiratory acidification (p≥0.356).


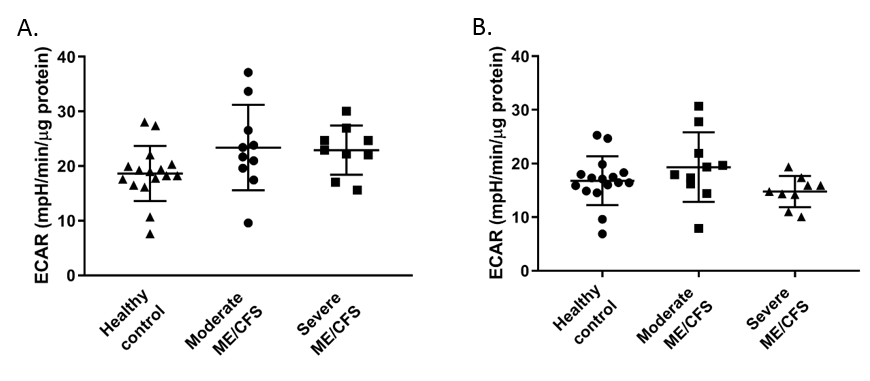


**Figure S2. Glycolytic capacity** in PBMCs from healthy controls (n=16), moderate ME/CFS (n=10), and severe ME/CFS patients (n=9). (A) Glycolytic capacity as calculated from a glycolysis stress test following the manufacturer’s protocol. (B) Glycolytic capacity adjusted for respiratory acidification. Groups were compared using a one-way ANOVA with post-hoc Bonferroni correction.


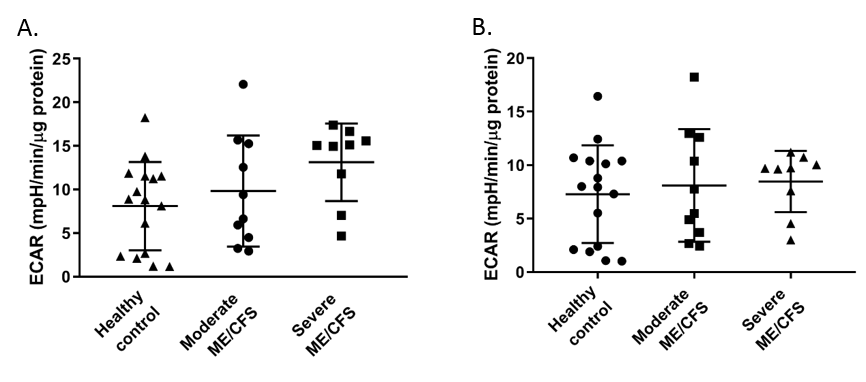


**Figure S3. Glycolytic reserve** in PBMCs from healthy controls (n=16), moderate ME/CFS (n=10), and severe ME/CFS patients (n=9). (A) Glycolytic reserve as calculated from a glycolysis stress test following the manufacturer’s protocol. (B) Glycolytic reserve adjusted for respiratory acidification. Groups were compared using a one-way ANOVA with post-hoc Bonferroni correction.


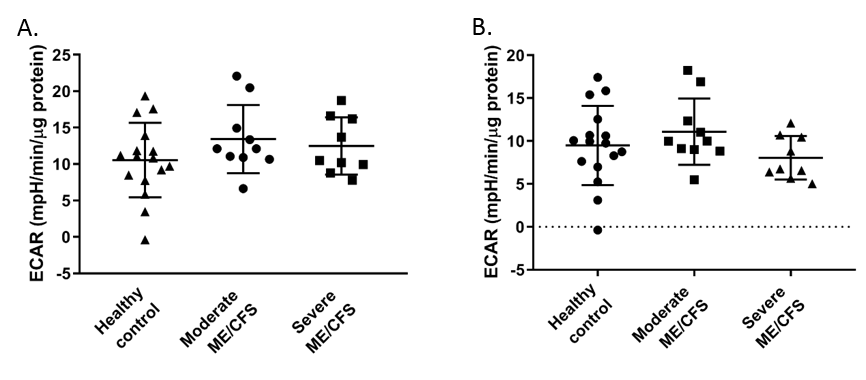


**Figure S4. Non-glycolytic acidification** in PBMCs from healthy controls (n=16), moderate ME/CFS (n=10), and severe ME/CFS patients (n=9). (A) Non-glycolytic acidification as calculated from a glycolysis stress test following the manufacturer’s protocol. (B) Non-glycolytic acidification adjusted for respiratory acidification. Groups were compared using a one-way ANOVA with post-hoc Bonferroni correction.
