# Supplementary material for: The effect of myalgic encephalomyelitis/chronic fatigue syndrome (ME/CFS) severity on cellular bioenergetic function
Source: PLoS One. 2020 Apr 10;15(4):e0231136. doi: 10.1371/journal.pone.0231136 (PMC7147788; doi:10.1371/journal.pone.0231136)
Supplement: Attachment — Submitted filename: Response to reviewers.docx [file pone.0231136.s003.docx]

William Leech Building
Institute of Cellular Medicine

Newcastle University
Newcastle upon Tyne
NE2 4HH, UK

cara.tomassmith@nhs.net

Jianhua Zhang

Academic Editor

PLOS ONE

21^st^ February 2020

Ref: PONE-D-19-34254

Manuscript title: ‘The effect of Myalgic Encephalomyelitis/chronic fatigue syndrome (ME/CFS) severity on cellular bioenergetic function’

Authors: *Cara Tomas, Joanna Elson, Victoria Strassheim, Julia Newton and Mark Walker.*

Dear Dr Zhang

We would like to thank you for your time in considering this paper for publication. Please find attached the revised manuscript entitled ‘The effect of Myalgic Encephalomyelitis/chronic fatigue syndrome (ME/CFS) severity on cellular bioenergetic function’ PONE-D-19-34254 for your consideration.

We would like to sincerely thank all three of the reviewers for their comments. We have addressed both the major and minor comments raised by the reviewers. Substantial changes have been made including additions to the introduction to better reflect the literature surrounding the techniques utilised in the manuscript. The methods section has been altered to better describe how cells were isolated, the experimental technique of extracellular flux analysis, and to describe more clearly the accepted parameters for determining disease severity in ME/CFS patients. This has been an area of intense debate. A new figure and table have been added to the manuscript to reflect analysis that has been completed following a suggestion from reviewer 3. This added analysis looks at the relationships between mitochondrial stress test parameters in the three cohorts. The discussion and conclusions have also been amended after considering comments from the reviewers.

Changes are itemised below it italics and are highlighted in the track changes version of the manuscript.

**Reviewer #1:**

1) As the authors state, the heterogeneity of this disease is wide-ranging. As such, the line between moderate and severe forms of this disease would seem to need to be better defined aside from just the ability to come into the fatigue clinic. Perhaps a more thorough description in the methods/results as to the criteria chosen (including those outlined in the NICE guidelines), and how these criteria might influence how and when PBMCs were isolated (i.e. age, sex, stage of disease) would all provide critical information that is missing.

***Author’s comment:*** *We thank the reviewer for their comment. We have now amended the methods section to make the differences between the disease severity groups clearer.*

2) Claiming that the severe group significantly delineates from the moderate group (i.e. in Figure 2B) seems to also be attributable to a few of the more severe cases, as ~40% of individuals still cluster in a similar range to the moderate cohort. Along these lines, there also is some bias towards to the severe cohort, as 25 patient PBMCs fall into the severe group, whereas only 13 fall into the moderate group. As such, it is hard to fully determine which of the 25 individuals is the best representation of this cohort.

***Author’s comment:*** *We have now revised to text to more accurately reflect the data by stating that it is only a sub-section of the severely affected cohort that shows the decrease in glycolytic acidification compared to the moderately affected cohort.*

3) The difference between glycolytic acidification and respiratory acidification, and how they are determined, is also not made very clear. A better description of how the authors came to differentiate between the two is needed.

***Author’s comment:*** *We have added a section to the text to clarify the calculation of respiratory and glycolytic acidification.*

**Reviewer #2:**

Major points:
1) The innovation is not clear since prior studies have underlined both glycolytic and mitochondrial dysfunctions as contributors to the illness. How is this study contributing in a better understanding of ME/CFS physiopathology? The citations undervalue prior works related to the approach in general (eg: Shiva, Darley-Usmar, Molina groups among others).

***Author’s comment:*** *We have added sections to the introduction and conclusion to outline the importance of this work, particularly the inclusion of severely affected patients in the study.* *We have also updated the introduction and discussion to better reflect prior work in this area.*

2) The authors have highlighted in the discussion the importance of repeating these experiments on other cell types. My question is along those lines, given that PBMCs are metabolically quiescent cells; how are these findings relevant to the disease mechanisms? Are the authors confident that their findings would still be pertinent in metabolically active cells?

***Author’s comment:*** *We thank the reviewer for their comment. As we showed in our previous publication with this data-set, PBMCs from ME/CFS patients are shown to have significantly different bioenergetic profiles than those of healthy controls. While PBMCs may not be the most metabolically active cells, the fact that we have shown there to be differences between the way in which ME/CFS PBMCs and healthy control PBMCs utilise glucose shows their importance. This preliminary work is vital for building an overall picture of fuel utilisation in ME/CFS, which will ultimately include different cell types. PBMCs are more easily accessible than most other cell types, which is why the initial study utilised their availability. Moving forward, we will look at fuel utilisation of other cell types in order to see if the differences seen in PBMCs can also be identified in other tissues.*

3) The authors have focused their study primarily on mitochondria respiration rates and glyscolysis; and have switched the cells to low glucose to assess the effect of glucose concentration rates on ATP production. Have the authors considered studying the lipid metabolism? Are mitochondria reliant on fatty acid beta-oxidation in this model? If so, is this response also different between the two ME/CFS Cohorts? This limitation should be addressed or at least discussed.

***Author’s comment:*** *As this was a re-analysis of the previously published data that did not look at lipid metabolism, therefore lipid metabolism was not included in this manuscript. We are aware of the importance of lipid metabolism in ME/CFS that has been reported in a number of manuscripts looking at metabolism in those with ME/CFS. Other studies we have conducted have looked at this aspect of ME/CFS cellular bioenergetic function but were not included in the scope of this manuscript. We have added a section to the discussion to address this limitation.*

4) The authors suggest that low glycolysis rates may explain the severity of the disease in the severely affected group. If that is the case, the study and its conclusion would benefit from modeling altered glycolysis function (pdh mutations, PDH silencing) in moderately affected cells to see if it mimics disease severity.

***Author’s comment:*** *As discussed previously, the purpose of this study was to assess the previously published data using new techniques. This manuscript describes exciting new findings including the ability of severely affected patients to utilise the glycolysis pathway and mitochondria for ATP production.*

**Reviewer #3:**

Major points
1) A comprehensive multi variate analysis is important since it may reveal changes in the overall bioenergetic program as shown in Mitochondria in precision medicine; linking bioenergetics and metabolomics in platelets. Redox Biol 22, 101165 2019 An advantage of this approach is that you do not need to separate into moderate or severe which increases statistical power-which maybe the real reason you do not see a relationship to disease severity. You can also include a clinical parameter in the analysis to test this.

***Author’s comment:*** *We have looked at the reference you recommended and completed the types of analysis described in the paper. We have added another figure and table to the manuscript to show the newly analysed data (figure 2). The new figure shows the most significant correlations with the graphs for all other correlations can be seen in the supplementary data file. We have also added a section to the paper to describe the newly conducted analysis.*

2) A BHI calculation may also be revealing here.

***Author’s comment:*** *We thank the reviewer for their suggestion and this is something we considered including, however, 13 of our samples have a negative reserve capacity, which means that a BHI cannot be calculated. We were reluctant to embark on new analysis that would require discarding this number of samples.*

3) Do not understand Figure 3 -The with and without glucose experiment is confusing. Some clearer description of the original experiment is needed so readers do not need to read both. Some justification of using frozen cells is needed-the mitochondria cannot make ATP after freeze thawing so is the valid?

***Author’s comment:*** *We thank the reviewer for their comment regarding the lack of clarity surrounding figure 3. The figure shows the glycolysis of cells as reported in the previous manuscript (Fig 3A) and after that, data has been adjusted for glycolytic acidification. We have amended the text to make this clearer and given a brief description of how glycolysis was measured in the previous publication.*

*Our previous publication investigated the effect of freezing on PBMC mitochondrial function and found “the absolute values of some of the parameters are affected by the freezing process; [however] the pattern can be seen in both cohorts and therefore either fresh or frozen samples can be used to detect differences between control and CFS cohorts.” We also concluded, “frozen samples are adequate to show the differences between the cohorts sufficiently”. Given that the effect of freezing is something that we considered very carefully in our original study, we are confident that our results from frozen cells are valid.*

Minor Points
1)PBMC are a heterogenous mixture of cells with different bioenergetic programs (see Redox Biology 2:206-210 2014). This limitation needs to be noted.

***Author’s comment:*** *We have added a comment addressing this in the discussion.*

2) The citations are woefully inadequate and do not reflect the current thinking in the field of translational bioenergetics or the significant contributions from other groups. Please extend the introduction and discussion significantly to address this.

***Author’s comment:*** *We apologise for the absence of key work within the field. We have updated the text to better reflect the key prior work.*

3) Materials and Methods need to be more complete defining the method for PBMC isolation and characterization even though this maybe in the previous publication.

***Author’s comment:*** *We have now given a more thorough description of this process in the methods section.*

4) Strictly speaking this is cellular respiration not mitochondrial since these are not isolated mitochondria and other factors contribute to the control of respiration which are not mitochondrial-please change this terminology. (see ref in comment 2). This is not ATP production but ATP linked respiration (see Biological chemistry 401 (1), 3-29 2019).

***Author’s comment:*** *We have amended the text to use the terminology suggested.*

5) A section is needed on normalization and the definition of parameters-eg coupling efficiency.

***Author’s comment:*** *This has been added to the methods section.*

6) The statistical markings are missing from 2A. Does not look significant but text says it is.

***Author’s comment:*** *We included the statistical markings on the graph for Fig 2A so we are unsure as to why they did not appear on the version seen by reviewer 3.*

7) No need for sentence 191 -this is not the guideline but the published papers have for some time shown this understanding of the different processes contributing to ECAR

***Author’s comment:*** *This sentence has been removed.*

8) The conclusions are not really conclusions but a list of what was done-rewrite to discuss conclusions

***Author’s comment:*** *The conclusions section has been re-written in order to be more focused on the conclusions that can be drawn from this work.*
